# Evaluating cardiovascular risk in an Inclusion Health population admitted to secondary care

**DOI:** 10.3389/fcvm.2025.1507029

**Published:** 2025-05-16

**Authors:** Yujia Gao, James Norman

**Affiliations:** ^1^ UCL Medical School, University College London, London, United Kingdom; ^2^ Division of Infection, University College London Hospitals NHS Foundation Trust, London, United Kingdom

**Keywords:** Inclusion Health, QRISK3 scores, cardiovascular risk, CHIPA, marginalised populations

## Abstract

**Introduction:**

Inclusion Health (IH) encompasses individuals facing social exclusion, presenting a myriad of risk factors leading to compromised health. IH populations, including homeless individuals, substance abusers, and vulnerable migrants, have higher disease prevalence, earlier mortality, and more barriers to healthcare access, with increased risks of developing cardiovascular diseases. While QRISK3 is a validated tool for predicting ten-year cardiovascular risks in the general population in primary care, its application in secondary care or IH populations remains underexplored.

**Methods:**

This prospective study, conducted at University College London Hospital (UCLH) between December 2021 and December 2023, evaluated IH patients using a locally-developed Collaborative Holistic Inclusion Health Patient Assessment (CHIPA) tool, incorporating clinical scoring systems including QRISK3. QRISK3 scores and corresponding Q ages were calculated independently using its online platform, based on patient demographics, cardiovascular histories, and cholesterol levels. The CHIPA was a detailed medical and social history questionnaire used to evaluate the IH patients referred to IHT in a consistent manner, including the incorporation of questions related to QRISK3 and other scoring systems.

**Results:**

A total of 217 IH individuals were included in this study (median: 48 years; range: 25-81 years). Our IH patient cohort exhibited significantly higher QRISK3 scores compared to the general population (p < 0.001, paired Wilcoxon test), ranging from 0.1% to 81.7%, with an average increase of 8.6%. The corresponding Q age was, on average, 13 years older than patients' actual age, with a median of 62 years, and 35% of patients had a positive cardiovascular history. The relative risk ranged from 0.5 to 78.0, with an average of 4.0. Compared with the general population, IH patients demonstrated elevated cardiovascular risk.

**Discussion:**

We highlight the utility of QRISK3 and CHIPA tools in early cardiovascular risk identification within the Inclusion Health cohort of patients, facilitating timely interventions. Integrating clinical risk scores into holistic assessments can improve referral practices, potentially enhancing overall health outcomes and reducing mortality in IH patients.

## 1 Introduction

Inclusion Health (IH) is a comprehensive term denoting individuals subjected to social exclusion, often encountering a convergence of diverse risk factors contributing to compromised health, including economic deprivation, exposure to violence, stigma, and complex trauma (1). IH pertains to various marginalised groups, such as those facing homelessness, substance use, vulnerable migrants, Romani people and Traveller communities, sex workers, and individuals within the criminal justice system (2). These individuals exhibit significantly raised disease prevalence, spanning cardiovascular and respiratory conditions as well as infections and mental health disorders (3, 4). Their increased vulnerability to physical, mental health, and substance misuse is recognised as tri-morbidity, which is associated with markedly poorer health outcomes (5).

Conversely the IH population tend to encounter suboptimal healthcare with a lack of adherence to standard screening pathways. Barriers to healthcare include a combination of stigma, negative previous experiences of healthcare, a lack of flexibility from specialist services, mainstream services being unaware of or unable to meet Inclusion Health patient needs, Inclusion Health groups experiencing competing needs and priorities, and service fragmentation (6). Access to all community services necessitates proof of connection to a chosen locality, rendering it unattainable for individuals experiencing homelessness (6, 7). Barriers to primary and preventive care contribute to an increased frequency of medical emergencies and Emergency Departments (ED) attendances: Homeless individuals in England attend emergency departments three times more frequently than those without homelessness experiences, resulting in a four-fold risk of unplanned hospital admission and three times longer hospital stays (7–9).

A combination of increased health needs and reduced access to healthcare results in IH patients having markedly inferior health outcomes and higher mortality rates compared to the general population. In the UK, where the average life expectancy was 81.77 years in 2023, individuals experiencing homelessness and rough sleeping faced an average age of death as low as 43 years old for women and 45 years of age for men (1). The all-cause mortality was twelve times higher in females in IH groups and eight times higher in males, highlighting pronounced health disparities (3).

As a growing field IH medicine aims to address the social and health inequities that disproportionately affect marginalised populations. Its main goals are to draw emphasis to the extent and effects of extreme inequality, advocate for preventive and early intervention strategies, enhance access to essential services for individuals adversely affected by social exclusion, and make efforts to include the patients in the planning and implementation of their medical and social care (1).

In 2021 University College London Hospital (UCLH) launched the Inclusion Health Team (IHT), led by a consultant physician with a background in Infectious Diseases (ID) and General Internal Medicine (GIM). This was an initiative to provide comprehensive clinical assessments of IH inpatients and address the unmet health and social needs of the admitted IH patient population. The Collaborative Holistic Inclusion Health Patient Assessment (CHIPA) tool was developed to evaluate patients referred to the team. As part of this tool, the team incorporated existing frameworks and validated clinical scoring systems [QRISK3, FRAX, and the Malnutrition Universal Scoring Tool (MUST)] related to morbidity and mortality for comparative analysis with literature on the general population.

Given the challenges with arranging access to General Practice, scores like QRISK3 and FRAX were adopted to try and evaluate the chronic risks of common health problems, such as cardiovascular disease, in this population. The QRISK3 risk calculator is the standard care of risk tool for prediction of ten-year risk for cardiovascular events in England (10) and is validated in primary care among the general population. While individuals facing homelessness have an approximately three times greater risk of developing cardiovascular disease and heightened cardiovascular mortality (11), the use of QRISK3 in secondary care or in IH populations is poorly investigated.

This study aims to use the QRISK3 score to assess the cardiovascular risk in IH populations admitted to UCLH, compared to the general population. The objective is to contribute to the ongoing progress of IH medicine by highlighting the significance of cardiovascular risk assessment as a risk factor for early mortality. The study also aims to provide insights for the future enhancement of integrated treatment approaches tailored for IH groups, thereby addressing the unique healthcare needs of this population.

## 2 Materials and methods

This prospective single-centre study was conducted on IH patients referred to and seen by IHT at UCLH in London between December 2021 and December 2023. All patients had been admitted via the Emergency Department or Ambulatory Care Unit and were inpatients within UCLH. The study utilised the CHIPA framework developed within the multidisciplinary team, incorporating various history taking elements alongside clinical scoring systems. These included cognitive assessments (Abbreviated Mental Test Score – AMTS) and established tools such as QRISK3, FRAX, and MUST as surrogates for risk of morbidity and mortality. All patients provided consent for data collection. The only exclusions were based on age, the QRISK3 is not validated in patients less than 25 years of age for example, or lack of data (such as height and weight), as per the requirements to complete the QRISK3. As such we do not anticipate any bias from the perspective of the authors.

The QRISK3 scores utilised in our study were independently computed via the online tool available at https://qrisk.org/ and was not modified in anyway. Data was inserted into the QRISK3 calculator using components of each patient's past medical history, such as history of smoking, incorporated as standard into the CHIPA, up-to-date cholesterol to high density lipoprotein testing, acute blood pressure monitoring, and the patient's height and weight, along with their postcode if they had accommodation. The QRISK3 risk calculator was able to generate a percentage estimate of ten-year risk of heart attack or stroke, the equivalent percentage estimate of ten-year risk for a healthy person (defined as no adverse clinical indicators and a cholesterol ratio of 4.0, a stable systolic blood pressure of 125, and body mass index of 25) of the same age, sex, and ethnicity, a relative risk between the two percentage risks, and an estimated “healthy heart age” for the patient. The “healthy heart age”, or “Q age”, is defined as the age at which a healthy person of the same sex and ethnicity as an individual would be expected to have their equivalent 10-year QRISK3 score.

### 2.1 Patient and public involvement

Patients were not involved in the design and conduct of this research. During the feasibility stage priority of the research question, choice of outcome measures, and methods of recruitment were informed by discussions with patients during their admission with the IHT.

### 2.2 Statistical analysis

The estimated QRISK3 scores in the IH cohort and the expected QRISK3 scores from the general population were compared using paired t-tests and paired Wilcoxon test. A significance level of *p* < 0.05 was applied throughout.

## 3 Results

A total of 223 patients were identified and underwent a CHIPA consultation (Table 1). QRISK3 scores were successfully computed for 217 patients, with 1 patient excluded due to an absence of vital basic details such as height and weight and 5 patients excluded due to age below 25. Initial IH assessments were primarily conducted by IH consultants (72%), IH specialist nurses (24%), and doctors on consultant supervised “taster day” experiences (3.2%). The majority of patients were male (78%), with a median age at admission of 48 years and a mean age of 49 years (range from 25 to 84 years). The predominant ethnicity was UK born Caucasian (60%), and 98% of patients had no prior consultations with the IH physicians.

**Table 1:** Patient demographics and clinical details upon referral.

Age	Median: 48 years; range: 25–84 years		
Gender	Male	170	78%
	Female	47	22%
Ethnicity	White United Kingdom	131	60%
	White Irish	16	7%
	White Gypsy, Irish Traveller, or Roma	3	1%
	Any other white background	18	8%
	White and black Caribbean	2	1%
	White and black African	1	0%
	White and Asian	2	1%
	Any other mixed or multiple ethnic background	0	0%
	Indian	3	1%
	Pakistani	1	0%
	Bangladeshi	1	0%
	Chinese	3	1%
	Black African	10	5%
	Black Caribbean	2	1%
	Any other black, black British, or Caribbean	10	5%
	Arab	10	5%
	Any other	4	2%
IVDU	No	204	94%
	Active use	11	5%
	Ex-user	1	0%
	Skin-popping	1	0%
CADU	No	181	83%
	Yes	36	17%
Alcohol misuse	No	177	82%
	Yes	40	18%
Alcohol/Other withdrawal	No	193	89%
	Yes	14	6%
	Suspected	6	3%
	Previous/risk	4	2%
Seen by IHT before	No	212	98%
	Yes	5	2%

IHT, Inclusion Health team; IVDU, intravenous drug user; CADU, crack cocaine users.

**Figure 1:**
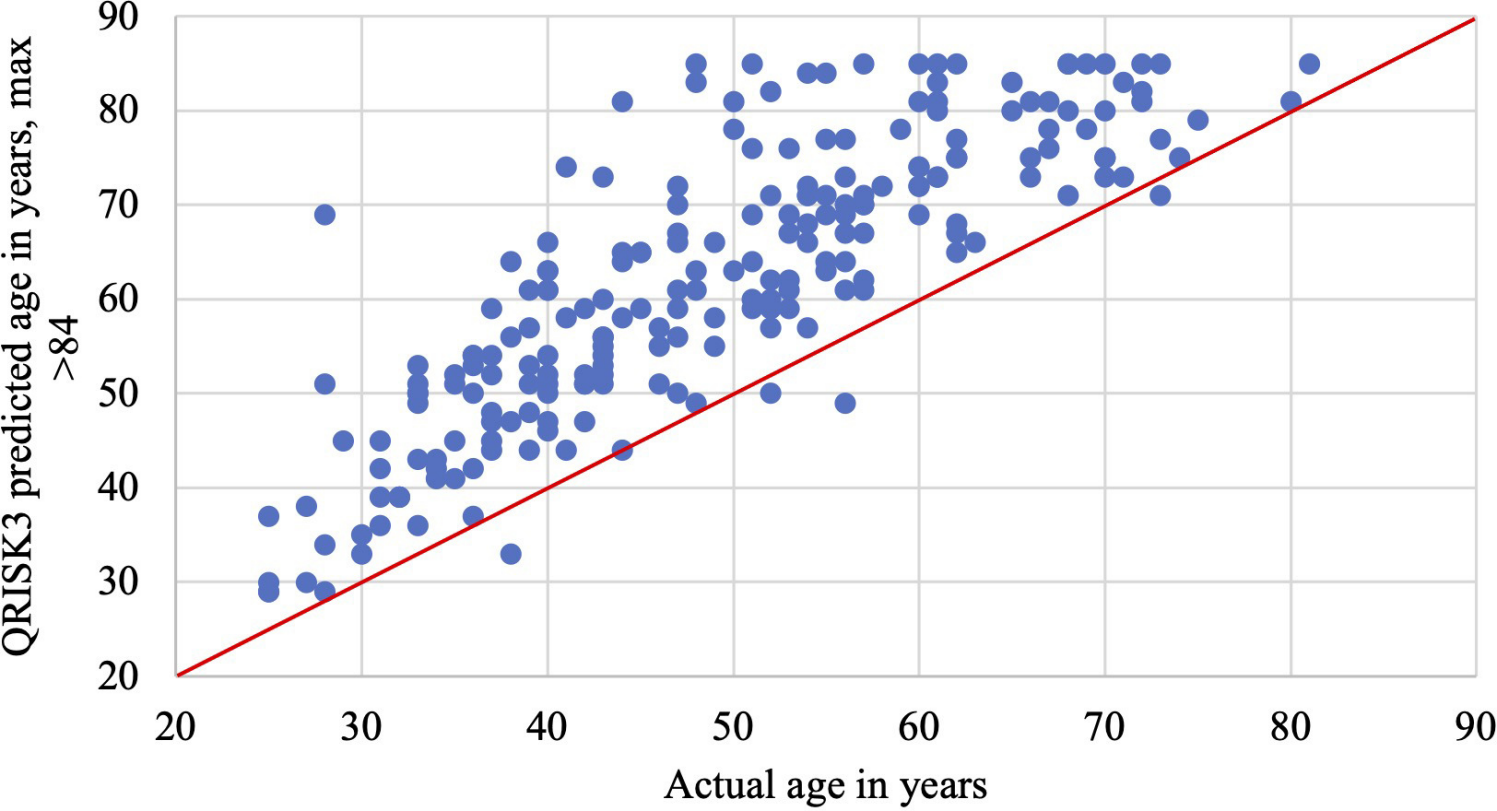
Age versus QRISK3 predicted age in years: the maximum QRISK3 predicted age is >84 years, which was recorded as 85 years on this graph.

**Figure 2:**
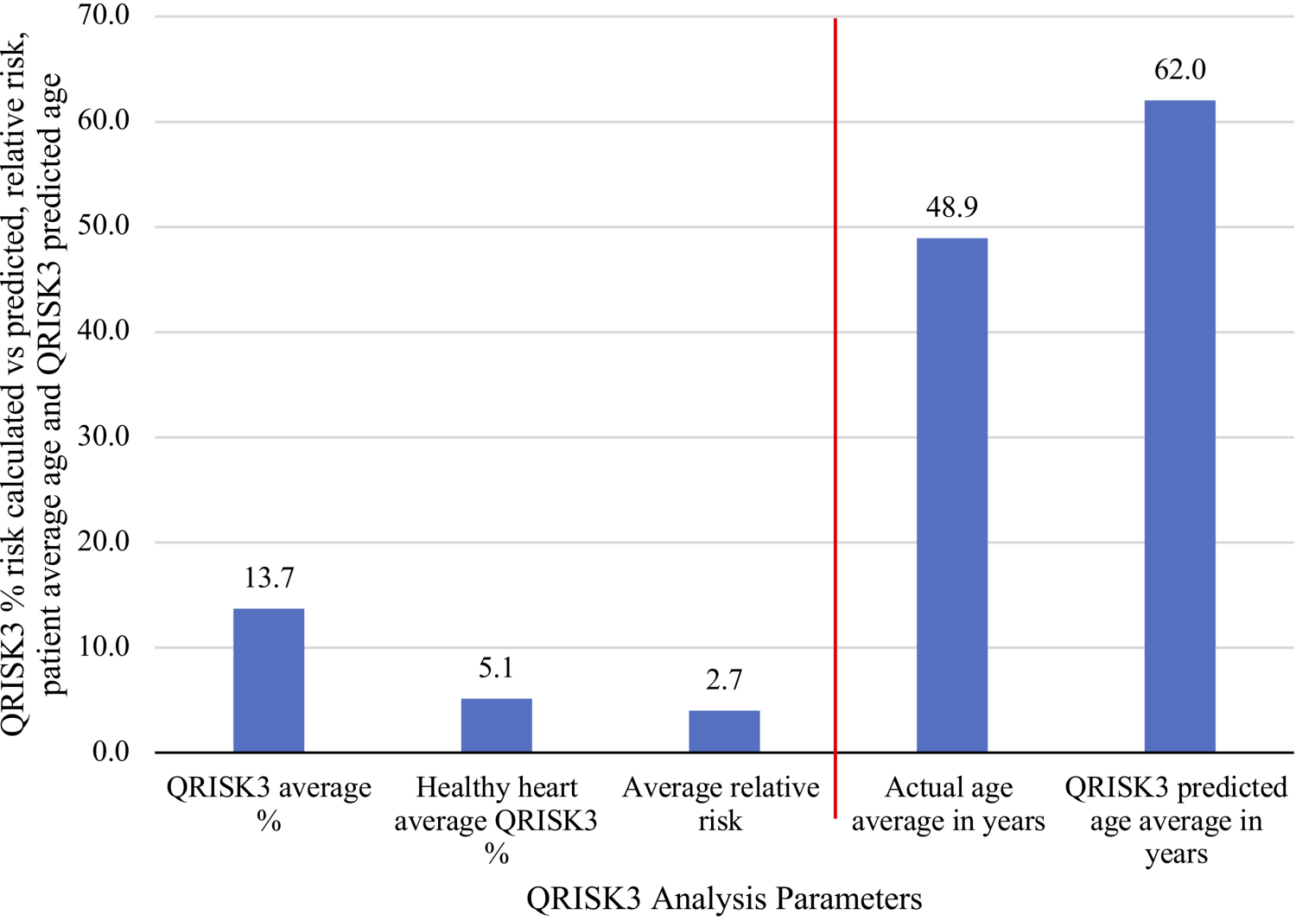
Comparing average results for QRISK3 analysis, including relative risk, versus patients’ average actual and predicted ages.

77 patients were identified as having a history of cardiovascular disease, with 34% having hypertension, 14% ischemic heart disease, and 9% atrial fibrillation (Table 2). The median QRISK3 score was 8.5%, with a mean of 13.7% ± 13.7% (standard deviation) (Figure 2). When compared to the general population, the expected QRISK3 scores at equivalent ages ranged from 0% to 33%, indicating statistically significant differences (*p* < 0.001, Wilcoxon signed rank test). Corresponding Q ages, based on observed QRISK3 scores in our patient cohort, were significantly higher than patients' actual ages (*p* < 0.001, Wilcoxon signed rank test) (Figure 1). The relative risk ranged from 1 to 78, with an average of 4 (Table 3; Figure 2).

**Table 2:** Cardiovascular history and presenting complaint.

Cardiovascular condition		Past medical history count (*n* = 77)	Presenting complaint count (*n* = 49)
Atrial fibrillation		7	Arrythmia: 8
Bradyarrhythmia		2	
Palpitations unknown cause		1	
Tachyarrhythmia		1	
Postural Tachycardia syndrome		0	
Unspecified arrythmia		1	
Congestive/left heart failure		4	Heart failure: Acute: 2 Acute on chronic: 3
Right ventricular failure		2	
Heart failure excluded on previous echocardiogram		1	
Cardiomegaly		1	N/A
Hypotension, including postural		2	
Hypertension, including secondary causes		26	
Complications of hypertension		3	
Angina		3	1
Ischemic heart disease		11	N/A
Congenital heart disease		2	
Previous cardiac arrest		2	
Previous percutaneous coronary intervention		1	
Previous valve surgery		0	
Permanent pacemaker		1	
Implantable cardioverter-defibrillator including removed as malfunctioning		2	
Reveal device		1	
Infective endocarditis (IE)		1 (resolved)	2
Septic emboli		1	N/A
Pleural effusion		n/a	4
Electrocardiogram (ECG) abnormalities	Prolonged QTc		21
	T wave inversion		1
	Right bundle branch block		2
	Possible acute coronary syndrome		1
	Non-specific		2
	Abnormal but unchanged from previous		1
	Abnormal but likely spurious		1

QTc, QT corrected for heart rate.

**Table 3:** QRISK3, Q age and relative risk.

Metric	QRISK3% score	QRISK3 “healthy person” % risk	Actual age	QRISK3 ‘healthy heart’ age	Relative risk
Min	0.1	0.1	25.0	29.0	0.5
Max	81.7	33.4	81.0	85.0	78.0
Median	8.5	2.9	48.0	63.0	2.7
Mean	13.7	5.1	49.1	62.0	4.0

QRISK3% score threshold^a^	**≥7.5**	**≥10**	**≥20**
Count (%)	117 (54%)	96 (44%)	55 (25%)

^a^QRISK3% score thresholds indicate the percentage of patients with a 10-year cardiovascular risk at or above the specified cutoff (≥7.5%, ≥10%, ≥20%), calculated from the QRISK3 online tool: https://qrisk.org/. Higher thresholds reflect greater predicted cardiovascular risk.

Same admission follow-up with the IHT was conducted for 125 patients, and 31 patients passed away within the timeframe of the study, including 5 deaths during their admission when seen by IHT. The duration from the initial IHT review to death varied from 6 to 599 days, with an average of 274 days. The causes of death were predominantly unknown, although one patient experienced an out-of-hospital cardiac arrest. Within our patient cohort the mean age of death was 57 and the median was 59 (range 29–81). These findings underscore the diverse age range and complexities associated with mortality within the studied population. A single patient was identified as having had an inpatient non-ST elevation myocardial infarction (NSTEMI), a diagnosis made by the IHT. This patient was managed with percutaneous coronary intervention (PCI) at UCH's local tertiary referral centre, with a good outcome.

## 4 Discussion

Our study conducted a direct quantitative comparison of the cardiovascular risk within our IH patient cohort in relation to the general population, employing the QRISK3 score. Figure 2 summarises our primary take-home messages of this study. Notably IH patients seen by UCH IHT between December 2021 and 2023 demonstrated a median relative risk of 2.7 in developing cardiovascular events in the next ten years according to their QRISK3 scores, with a significant cardiovascular age discrepancy of 13 years when compared to the general population (Figure 2).

In the UK, where the average life expectancy was 81.77 years in 2023, individuals experiencing homelessness and rough sleeping faced an average age of death as low as 43 years for women and 45 years for men (1). Our study revealed a comparable mean age of death at 57 years for our cohort of IH patients admitted to UCH within the timeframe of the study. Despite the small size of the death cohort and the predominantly unclear causes of death, this observed heightened cardiovascular risk within the IH population raises concerns about its potential contribution to premature mortality. Indeed, other studies have found cardiovascular disease as an important contributor to excess morbidity and mortality in Inclusion Health populations, including homeless populations, incarcerated populations, and people who use drugs (3, 12–14).

QRISK3 scoring provides a relative risk score that is benchmarked against the general population's heart disease risk and it is independent of current presenting complaint of the patient. It is worth considering its universal application for Inclusion Health patients during admission, given their known difficulties accessing and attending scheduled appointments in primary and secondary care. This approach may provide valuable insights into cardiovascular risk profiles, enabling proactive and targeted interventions to address the cardiovascular risks in this vulnerable population, from screening and preventive measures to acute and chronic management, incorporating early medical interventions and lifestyle modifications.

Data about individual types of cardiovascular disease (CVD) within the IH population remain limited. Our study demonstrated that one-third of our IH cohort had a cardiovascular history of hypertension, a condition which is typically managed in primary care settings to which many of these patients have limited access. For instance, homeless individuals and those with substance use disorders are less likely to engage with primary care services, leading to underdiagnosis and undertreatment of chronic conditions (15).

This lack of access to preventive care exacerbates the progression of modifiable risk factors such as hypertension and hyperlipidaemia, particularly when poorly controlled, compounding the overall risk burned in IH populations. This underscores the need for further observational and interventional studies on CVD in the IH population to inform the development of dedicated IH teams, mobile healthcare units, and tailored care pathways and programmes (11, 16), especially in the community.

Further research is also required to determine whether specific CVD types are disproportionately prevalent in IH populations and whether social determinants of health further modify their risk profiles. For example there is limited data on the effects of lifestyle associated with IH populations, particularly related to diet and street drug use, and the effects these might have on long term cardiac and other risk factors. Using these current data as a benchmark, with ongoing review of the selected patients going forwards, we hope to cast some light on this area in the future. We also look at a broader IH population, compared to other studies that are focussed exclusively on homeless patients, for example.

Our study had also identified prolonged QTc intervals as the most common ECG abnormality observed in our IH patient cohort. Prolonged QTc is a known risk factor for ventricular arrhythmias and sudden cardiac death, and studies suggest that it may serve as an independent predictive marker for cardiovascular mortality (17, 18), particularly in vulnerable populations with underlying risk factors such as illicit drug use and electrolyte imbalances. Given that nearly all ED patients undergo ECG upon admission, further research is warranted to determine whether QTc prolongation represents an independent, early cardiovascular risk marker in the IH population. Monitoring of these patients QTc is also important given many patients will be started on methadone during their admission, which is known to prolong the Q interval (19).

Our study is subject to limitations. The QRISK3 score is validated for the general population in primary care settings (10) whereas the study group is an inpatient population who are therefore more likely to have cardiovascular diseases regardless of their IH health status. Therefore we are limited in the interferences we can make from this data. Additionally, while the QRISK3 score accounts for several risk factors, potential confounding variables such as socioeconomic status, access to healthcare and lifestyle behaviours, for example smoking, alcohol use and substance misuse, likely contribute to the observed elevated cardiovascular risk scores (20) and are not part of the QRISK3 scoring. Previous studies have illustrated that social determinants of health, including unstable housing, fuel poverty and financial insecurity, significantly influence cardiovascular outcomes (20, 21). This is reflected in the wide range of our relative risk (0.5–78), highlighting the heterogeneity of cardiovascular risk profiles among IH patients. Clinicians interpreting such broad relative risk ranges should consider both clinical risk factors and individual social circumstances when tailoring interventions.

Follow-up data was obtained for just over half of our patients and the follow-up period has been short. Achieving continuity in care is particularly challenging within the IH patient cohort, as primarily due to their challenges to accessing healthcare and common status of homelessness, preventing access to causes of death and further comprehensive cardiovascular risk assessment. Despite insights from reattendance data at our centre, undisclosed new admissions in other hospitals may introduce unexplored variables into our study. On top of this nonattendance, difficulty accessing healthcare, and a lower level of education among the participants may contribute to the masking of conditions such as diabetes, atrial fibrillation, silent myocardial infarction, and mental health problems. These factors could lead to an underestimation of our QRISK3 results, as these conditions may go unrecognised due to patient lifestyles and lack of awareness.

Notwithstanding these limitations our study has provided preliminary evidence affirming the significantly raised cardiovascular risks in IH patients, in keeping with previous literature. This underscores the utility of the QRISK3 and CHIPA tools in the assessment of IH patients upon admission, providing a holistic perspective. This proactive approach can enable clinicians to identify cardiovascular risks in IH patients at an earlier stage, presenting opportunities for timely and preventive interventions, for example antihypertensives or lipid-lowering therapies, as well as referrals to preventive cardiology services. This has the potential to save both time and costs associated with managing more complex medical issues that may otherwise arise at later stages. Furthermore, the incorporation of clinical risk scores, including QRISK3, within the CHIPA tool, could prompt and facilitate improved referral and engagement with appropriate community services for IH patients at an earlier stage, thereby increasing patient awareness. Such considerations may contribute to enhancing overall health outcomes and potentially reducing mortality in IH patients. Further research should explore the long-term effectiveness of such interventions in reducing cardiovascular events and mortality in IH populations with larger sample size. Additionally, alternative cardiovascuarl risk stratification tools, such as the Framingham risk score (22), Systematic COronary Risk Evaluation 2 (SCORE2) (23), Revised Pooled Cohort Equations (RPCE) (24), and World Health Organization cardiovascular disease (WHO CVD) (25) models may also be used in parallel to optimise risk assessment strategies in this high-risk population.

## 5 Conclusion

In conclusion our study has demonstrated that the IH patient cohort exhibits significantly higher QRISK3 scores, suggesting an advanced cardiovascular age compared to the general population, with a markedly elevated risk of detrimental cardiac events. The use of QRISK3 and CHIPA tools has the potential to facilitate improved referral practices and early interventions, offering opportunities for enhancing overall health outcomes in IH patients.

## Data Availability

The raw data supporting the conclusions of this article will be made available by the authors, without undue reservation.
